# Exploring the interplay between zinc‐induced protein dyshomeostasis and mitochondrial dysfunction using viscosity‐sensitive sensor

**DOI:** 10.1002/smo.20240047

**Published:** 2024-10-12

**Authors:** Xuan He, Jiaqi Li, Wenye He, Jia Zhai, Yu Wei, Xin Zhang, Baoxing Shen, He Huang

**Affiliations:** ^1^ School of Food Science and Pharmaceutical Engineering Nanjing Normal University Nanjing China; ^2^ Faculty of Science National University of Singapore Singapore Singapore; ^3^ Department of Chemistry Research Center for Industries of the Future Westlake University Hangzhou Zhejiang China; ^4^ Westlake Laboratory of Life Sciences and Biomedicine Hangzhou Zhejiang China

**Keywords:** FLIM, mitochondrial damage, protein aggregates, viscosity sensitivity, Zn^2+^ detection

## Abstract

Mitochondria are crucial sites for protein quality control within cells. When mitochondrial stress is triggered by protein misfolding, it can accelerate abnormal protein aggregation, potentially inducing various diseases. This study developed a cascade‐responsive sensor, named AggHX, to monitor the microenvironment of protein aggregation induced by zinc (II) ions and the accompanying mitochondrial dysfunction. The AggHX consists of two key components: (1) A Zn^2+^ recognition group for triggering a fluorescent enhance response, and (2) a near‐infrared BODIPY scaffold that detects viscosity changes in cell aggregation via HaloTag. This sensor's mechanism of action is elucidated through photochemical and biochemical characterizations. To further investigate the relationship between protein aggregation and mitochondrial homeostasis, we employ fluorescence lifetime imaging microscopy to assess viscosity changes in protein aggregates under intracellular Zn^2+^ stress. This research provides insights into the dynamic behavior and spatial distribution of protein aggregates and mitochondria, contributing to a deeper understanding of their physiological roles in cellular processes and potential implications in disease pathology.

## Graphical abstract

A Zn^2+^ and viscosity cascade‐responsive sensor with red emission, AggHX, has a viscosity response of fluorescence intensity and lifetime to quantify Zn^2+^‐induced protein aggregation. High‐resolution imaging of mitochondrial damage and protein aggregation was accomplished with the aid of dual‐sensor mode, revealing the mutual promotion of both. FLIM imaging describes the viscosity values of different protein aggregates.

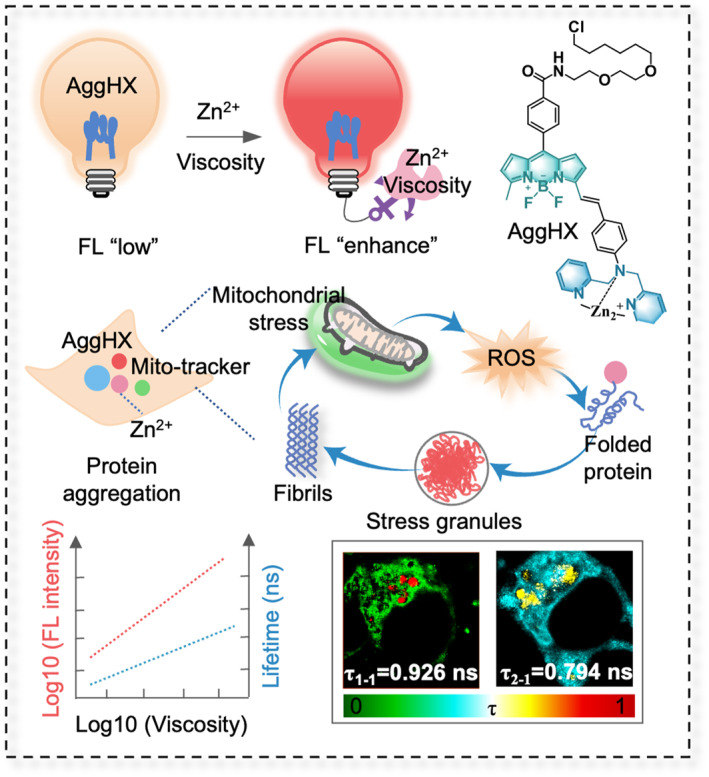

## 1 INTRODUCTION

Proteins are essential for cellular metabolism and functions, comprising the framework of cells and tissues. Most proteins fold into specific three‐dimensional structures, which are crucial for regulating physiological functions within the cell. Under certain stimuli, such as temperature changes, pH fluctuations, or the presence of metal ions, proteins could misfold and aggregate.^[1]^ These aberrant proteins may evade normal cellular clearance mechanisms, leading to disruptions in cell function, aging problems, and various human diseases.^[2]^

**FIGURE 1:**
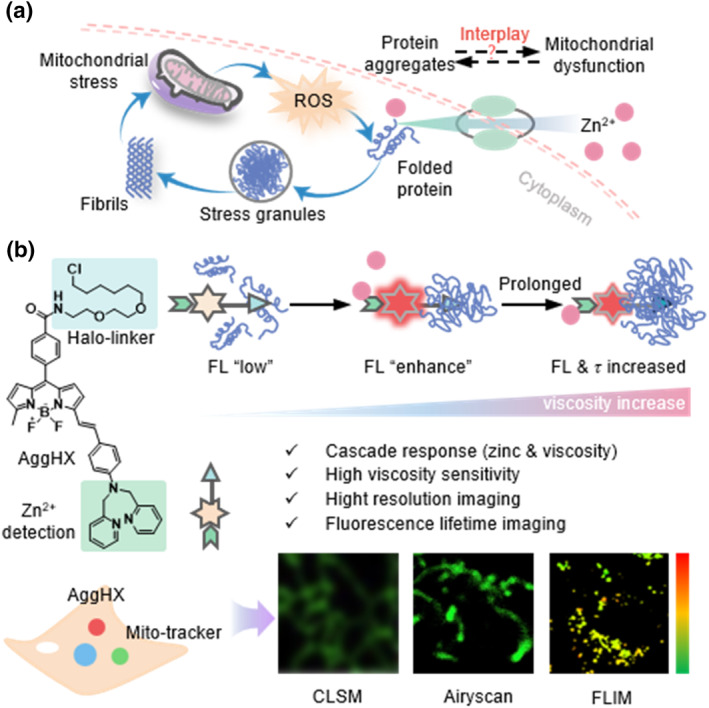
A dual cascade‐responsive fluorescent sensor (AggHX) sensitive to Zn^2+^ and viscosity for visualization of protein polymerization by high‐resolution and FLIM. (a) Interactions between protein aggregation induced by Zn^2+^ homeostatic imbalance and mitochondrial dysfunction. (b) The viscosity‐sensitive sensor (AggHX) with Zn^2+^‐induced fluorescence enhancement and mitochondrial localization dye for high‐resolution and FLIM imaging in living cells. FLIM, fluorescence lifetime imaging microscopy.

Zinc (II) ion, as an essential trace element for organisms, plays a vital role in physiological and cellular processes including growth, metabolism, DNA synthesis, and transcriptional regulation. Many studies have shown that zinc positively regulates cellular autophagy.^[3]^ When Zn^2+^ concentration reaches a certain threshold (20–200 μm), it promotes the production of reactive oxide species (ROS), leading to protein aggregation and mitochondrial dysfunction.[^4^, ^5^] It further exacerbates autophagy, and dysregulation of autophagy, in turn, disrupts Zn^2+^ metabolic homeostasis.[^6^, ^7^, ^8^] In brief, the Zn^2+^ accumulated to pathological concentrations can cause abnormal protein aggregation, disrupt mitochondrial homeostasis, and induce amyloidosis, which involves Alzheimer's disease, Parkinson's disease, and amyotrophic lateral sclerosis (ALS; Figure 1a). Therefore, developing visualization tools to observe zinc ion‐induced protein aggregation, as well as mitochondrial changes in number and function, is crucial for uncovering the underlying cellular mechanisms. These tools will provide effective research methods for developing diagnostic and therapeutic strategies for related diseases.

Fluorescent probes for quantitative detection have long been regarded as outstanding visualization tools, based on different chemical designs that are capable of detecting a diverse range of substances, including ROS, amino acids, metal ions and protein aggregates, and are suitable for cellular imaging.[^9^, ^10^, ^11^, ^12^, ^13^, ^14^] However, it is challenging to develop sensors capable of concurrently detecting metal ions and aggregated proteins. The difficulty primarily arises from the requirement to design sensors that could selectively report on zinc ions while simultaneously providing accurate measurements of dynamic changes in protein aggregation. Recent advancements have been made in the design of sensors for sensing protein aggregation through targeting local viscosity, polarity, and nucleophilicity. For instance, Liu et al. have developed a series of organic fluorophores that modulate the twisted intramolecular charge transfer. This approach allows for customizable sensitivity to environmental changes, facilitating the monitoring of β‐amyloid protein (Aβ) aggregation.^[15]^ Furthermore, Zhang and his research team have developed a dual‐functional BODIPY‐based molecular rotor sensor. It allows for the visualization of changes in protein aggregation viscosity within the cells by simultaneously detecting fluorescence intensity and fluorescence lifetime.^[16]^ Nevertheless, these sensors are limited by responding exclusively to alterations in the physical microenvironment of a single protein, and lacking the capability to concurrently detect multiple targets within the cell. Therefore, the advancement of sensors that will generate cascading responses to multiple intracellular targets is a critical technological challenge in achieving the research objectives.

BODIPY‐based sensors are characterized by high fluorescence quantum yields, photostability, and tunable absorption and emission wavelengths, demonstrating significant versatility for various applications.[^17^, ^18^] Herein, we have developed a dual‐responsive sensor (AggHX) based on BODIPY, which monitors the process of protein aggregation through changes in its fluorescence intensity and lifetime (Figure 1b). Meanwhile, by employing mitochondrial dyes for imaging, we can investigate the interaction between protein aggregation and mitochondrial damage. Dimethyl pyridine amine (DPA) is a widely used ligand for recognizing zinc ions, with the amine nitrogen serving as an effective electron donor. Therefore, when the AggHX sensor with a DPA structure binds to zinc ions, it would weaken the electron donating capacity of DPA, resulting in an increased fluorescence quantum yield and enhanced fluorescence. This change is accompanied by a slight blue shift in the spectrum from 674 to 615 nm.

Furthermore, AggHX is coupled to the protein of interest (POI) via HaloTag to identify aggregation‐prone proteins. Due to its notable viscosity sensitivity (*x*_AggHX_ = 0.75; ${x_{\text{AggHX}+\text{Zn}}}^{2+}$ = 0.43), the dynamic changes in viscosity during protein aggregation could be visualized effectively. In vitro experiments, the fluorescence intensity and lifetime of AggHX exhibit a quantitative linear correlation with the viscosity of the microenvironment. This dual‐mode response enables AggHX to quantify viscosity changes during protein aggregation within cells, thereby reflecting the progression of protein aggregation. Additionally, by analyzing mitochondrial number and imaging, we are able to assess mitochondrial damage. Above all, the fluorescent sensor developed in this study plays an effective role in quantifying Zn^2+^‐induced insoluble protein aggregation and reporting the aggregation process, making it a suitable imaging tool.

## 2 RESULTS AND DISCUSSION

### 2.1 AggHX sensor achieves specific selectivity for Zn^2+^

**FIGURE 2:**
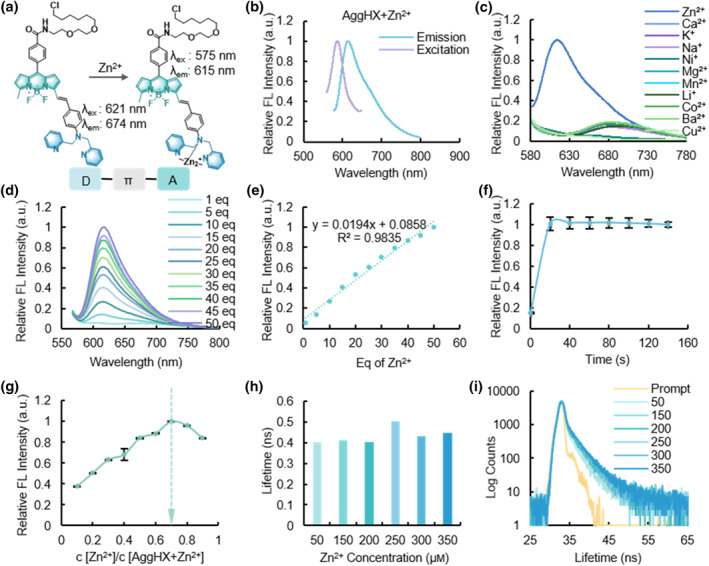
Selectivity of AggHX for Zn^2+^. (a) Fluorescence enhancement and spectrum blue shift of AggHX after complexation of Zn^2+^. (b) Fundamental excitation and emission spectra of Zn^2+^‐complexed AggHX. (c) Metal ion selectivity of AggHX. (d and e) Titration of Zn^2+^ concentrations. (f) Kinetics of Zn^2+^ titration. Error bars: standard error (*n* = 3). (g) Job plot curve of Zn^2+^ and AggHX. Error bars: standard error (*n* = 3). (h and i) Effect of Zn^2+^ concentrations on the fluorescence lifetime of AggHX.

Fluorescence sensors have been developed as effective tools for detecting specific metal ions in living organisms due to their advantages of simple operation and high sensitivity.[^19^, ^20^] And DPA group have been widely used in the design of zinc ion sensors.[^21^, ^22^] Building on this foundation, we employed the Knoevenagel condensation reaction to link the DPA group with the BODIPY fluorophore, resulting in the creation of a Zn^2+^‐induced, fluorescence‐enhanced red‐emitting sensor, designated as AggHX (Figure 2a, Supporting Information S1: Scheme S1–S4).

The nitrogen atoms in DPA as electron donors and the BODIPY fluorophore as a good electron acceptor were connected by a benzene ring as a bridge in the middle to form a typical sensor with D‐π‐A configuration. When the DPA is complexed with Zn^2+^, the electron pulling effect of the metal ions will make the electron donating capacity of DPA weakened, and thus fluorescence enhancement and spectral blue shift occur.^[23]^ After the complexation with Zn^2+^, AggHX exhibited significant shifts in its optical properties: the maximum excitation wavelength shifted from 621 to 575 nm, the maximum emission wavelength shifted from 674 to 575 nm, and the maximum absorption wavelength shifted from 618 to 582 nm (Figure 2b, Supporting Information S1: Figure S1a,b).

Further analysis of the fluorescence intensity changes of AggHX in the presence of various metal ions revealed that Zn^2+^ induced a substantial fluorescence enhancement. These findings underscore the selective sensitivity of AggHX towards Zn^2+^ (Figure 2c). In Zn^2+^ titration experiments, we incrementally increased the concentration of Zn^2+^ based on a 20 μm sensor. Across a wide range of Zn^2+^ concentrations (20 μm to 1 mm), the fluorescence intensity of AggHX maintained a linear relationship with Zn^2+^ concentration (Figure 2d,e). The detection limit of AggHX for Zn^2+^ was calculated to be 23.47 μm (Supporting Information S1: Figure S2a,b). By continuously increasing the equivalent (eq) of Zn^2+^, we could determine that the titration reached a plateau when the Zn^2+^ concentration was 120 times that of the sensor (Supporting Information S1: Figure S2c).

The fluorescence enhancement effect of Zn^2+^ on the AggHX sensor was a rapid process, with approximately 20 s sufficient to maximize the fluorescence intensity of the sensor (Figure 2f). This rapid response is likely attributed to the complexation of Zn^2+^ with the three nitrogen atoms on the DPA group. A Job plot curve was used to determine the stoichiometry of the metal‐ligand complex, where the peak indicated the complete coordination between the metal ion and the ligand. From the Job plot curve for AggHX and Zn^2+^, the optimal complexation ratio was determined to be 3:7 (Figure 2g). Therefore, in vitro experiments demonstrate that AggHX exhibits high selectivity and sensitivity toward Zn^2+^, making it an effective metal sensor that enhances its fluorescence intensity in the presence of Zn^2+^.

The fluorescence lifetime of a fluorophore is typically an intrinsic property, unaffected by factors such as excitation light intensity and fluorophore concentration. Instead, it is determined by the fluorophore's structure and the properties of its microenvironment, including polarity and viscosity.[^24^, ^25^] Accordingly, we measured the fluorescence lifetime of AggHX in the presence of different concentrations of Zn^2+^. The results showed that the difference in Zn^2+^ concentration had no significant effect on the fluorescence lifetime of the AggHX sensor, and the fluorescence lifetime was maintained at about 0.4 ns (Figure 2h,i).

### 2.2 Viscosity‐sensitive AggHX for detecting in vitro protein aggregation

Viscosity in the intracellular microenvironment varies significantly across different organelles and is closely related to cellular processes.[^26^, ^27^, ^28^, ^29^] BODIPY, a fluorescent molecular rotor, commonly quantifies viscosity in the microenvironment during protein aggregation in living cells. The AggHX molecule, featuring BODIPY as the backbone, has the electron donor (D) and the electron acceptor (A) portions, thus potentially allowing electron transfer. Destroying the sensor's D‐π‐A configuration effectively enhances its fluorescence intensity. In response to external disturbances, proteins misfold and aggregate, which are accompanied by changes in the microenvironment during this period, including a decrease in polarity and an increase in viscosity.[^30^, ^31^, ^32^]

**FIGURE 3:**
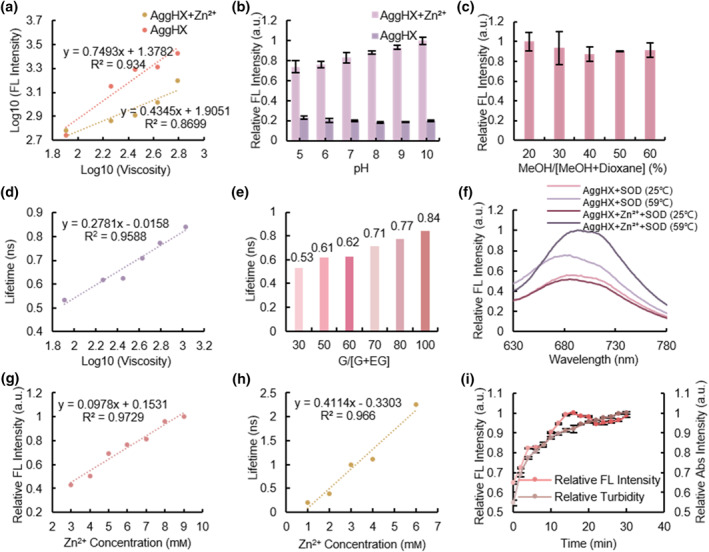
Detecting viscosity fluctuation through fluorescence intensity and fluorescence lifetime of AggHX in order to visualize protein aggregation in vitro. (a) The viscosity sensitivity of AggHX with and without complexed Zn^2+^ was determined separately in viscosity systems with different ratios of ethylene glycol/glycerol mixture. (b) The pH stability of AggHX/AggHX + Zn^2+^. Error bars: standard error (*n* = 3). (c) Different polar environments by adjusting the ratio of methanol/dioxane mixtures. Error bars: standard error (*n* = 3). (d) Linear fit of the logarithmic value of viscosity to the fluorescence lifetime of AggHX. (e) Fluorescence lifetime values of AggHX in different viscosities. (f) Relative fluorescence intensity of AggHX and SOD A4V‐Halo incubated with or without Zn^2+^, at 59°C or 25°C for 6 min. (g) Fluorescence response of AggHX for SOD A4V‐Halo aggregates induced by different Zn^2+^ concentrations. (h) Changes in fluorescence lifetime of AggHX during SOD A4V‐Halo aggregation induced by different Zn^2+^ concentrations. (i) Fluorescence and turbidity kinetics of SOD A4V‐Halo aggregation induced by 800 μm Zn^2+^. Error bars: standard error (*n* = 3).

To explore the viscosity sensitivity of AggHX, we measured the fluorescence intensity of the sensor, both with and without Zn^2+^ complexation, in different viscosity systems by adjusting the ratio of glycerol and ethylene glycol to simulate different viscosity environments. The viscosity sensitivity was calculated, indicating that the AggHX sensor was sensitive to viscosity, with the uncomplexed sensor being more responsive (Figure 3a). Given the common occurrence of microenvironmental changes during protein aggregation, we ensured that other factors, such as pH disturbances and polarity changes, did not interfere with the sensor's properties. The in vitro tests confirmed that the fluorescence emission of the Zn^2+^‐complexed sensor was significantly enhanced compared with the uncomplexed sensor, while the pH and polarity changes had minimal and negligible effects (Figure 3b,c). Additionally, supplementary anion interference experiments demonstrated that environmental anions did not affect the sensor's properties (Supporting Information S1: Figure S3a,b). Therefore, the AggHX was specifically sensitive to viscosity, as reflected by its fluorescence intensity and lifetime. The fluorescence lifetime of AggHX showed a great linear positive correlation with the logarithmic value of viscosity (*R* ^2^ = 0.96), enabling the quantification of local viscosity during protein aggregation both in vivo and in vitro (Figure 3d,e, Supporting Information S1: Figure S4a–f). This correlation allows the visualization of protein aggregation through the gradual increase in viscosity, which serves as a key indicator.

Temperature has long been recognized as a trigger for protein aggregation. In vitro experiments, we induced protein aggregation by heating the SOD A4V‐Halo mutants to 59°C. The Halo linker in the AggHX forms a highly specific covalent bond with the Halo Tag fusion tag added on the SOD A4V‐Halo mutants, enabling the recognition of A4V protein aggregates by AggHX.[^33^, ^34^] We observed a significant increase in the fluorescence intensity of AggHX after heating the SOD A4V‐Halo at 59°C for 6 min, with an even greater enhancement following Zn^2+^ complexation (Figure 3f). This response may result from the AggHX's cascade sensitivity to both Zn^2+^ and the high‐viscosity environment within protein aggregates. As the temperature increased from 25 to 59°C, the sensor's fluorescence intensity sharply rose at 45.8°C, indicating the onset of insoluble SOD A4V aggregates, which reached their maximum formation between 57.9 and 59°C (Supporting Information S1: Figure S5). These results confirm that protein aggregation is accompanied by an increase in viscosity.

In addition to temperature, Zn^2+^ at a certain concentration can promote the formation of protein aggregates both in vivo and in vitro. The extent of protein aggregation was described by the size of the aggregates. Previous experiments showed that at 120 eq (2.4 mm) of Zn^2+^, the fluorescence intensity of 20 μm AggHX reached a plateau (Supporting Information S1: Figure S2c). This suggests that for 20 μm AggHX, Zn^2+^ concentrations between 3 and 9 mm have no effect on the sensor's fluorescence intensity. Therefore, we applied Zn^2+^ concentrations in this range to induce the aggregation of SOD A4V‐Halo mutants in vitro. The result indicated a positive linear correlation between the sensor's fluorescence intensity and Zn^2+^ concentration (*R* ^2^ = 0.97) (Figure 3g). Since the Zn^2+^ concentration was ruled out as a factor influencing the AggHX fluorescence, we attributed the fluorescence enhancement to viscosity differences resulting from varying degrees of protein aggregation. In this case, Zn^2+^ induced protein aggregation and the degree of protein aggregation was positively correlated with a certain range of Zn^2+^ concentration. In glycerol viscosity experiments, we demonstrated that the AggHX's fluorescence intensity and lifetime were both proportional to viscosity. We further measured the sensor's fluorescence lifetime during protein aggregation induced by different Zn^2+^ concentrations (Figure 3h). The linear fit between fluorescence lifetime and Zn^2+^ concentration confirms that protein aggregation is indeed a process of increasing viscosity.

Furthermore, the kinetics of protein aggregation in vitro was characterized by tracking the trends in the AggHX's fluorescence intensity and turbidity during protein aggregation (Figure 3i). The fluorescence signal of the sensor provided real‐time insight into the dynamic process of its response to the POI, while the turbidity signal monitored the aggregation phase. Protein aggregation phases consisted of soluble oligomers formed early on and insoluble aggregates developed later. After approximately 15 min, the fluorescence reached its maximum, and the turbidity leveled off, indicating that the fluorescence response of the AggHX was consistent with the turbidity response of POI. These results suggest that AggHX is primarily used to detect the late stage of protein aggregation, specifically insoluble protein aggregates.

### 2.3 AggHX detects Zn^2+^‐induced protein aggregation in cells

**FIGURE 4:**
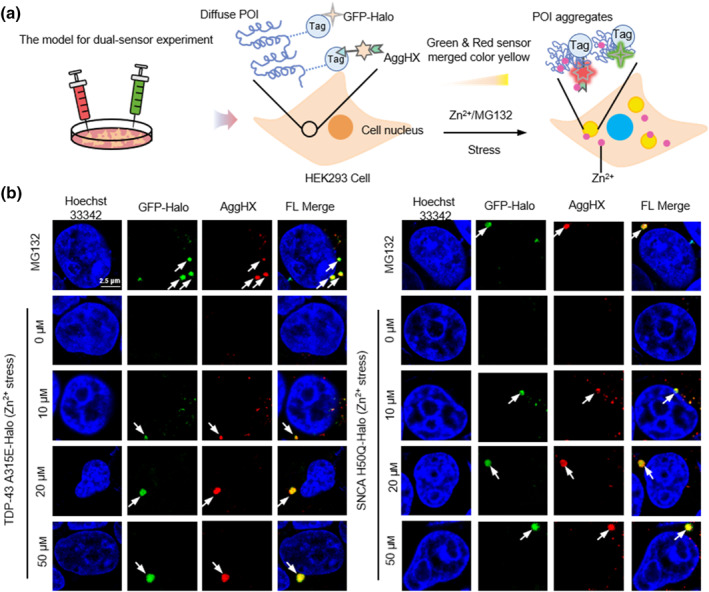
MG132 and Zn^2+^‐induced POI aggregates in HEK293 cells were labeled by AggHX sensor under the guidance of the AggTag method. (a) Schematic illustrating co‐localization of POI aggregates by green channel (GFP‐Halo) and red channel (AggHX) dual sensors. (b) TDP‐43 A315E‐Halo and SNCA H50Q‐Halo were transiently transfected in HEK293 cells and expressed, and the cells were incubated in 1 μm AggHX and 2 μm GFP‐Halo for 40 min. Without the influence of any interfering factors, the cells maintained a dark fluorescent background in red and green channels. Co‐localization of red and green channels was detected in cells, which were treated with 2 μm MG132 and 10, 20, 50 μm Zn^2+^, respectively. Scale bar: 2.5 μm. POI, protein of interest.

The long‐wavelength excitation of the AggHX sensor reduces the autofluorescence of endogenous chromophores and the damage to biological samples.^[35]^ Subsequently, we applied AggHX to live‐cell imaging. We chose a GFP sensor (*λ*_ex_ = 470 nm; *λ*_em_ = 529 nm), which operated in a different emission channel from AggHX (*λ*_ex_ = 575 nm; *λ*_em_ = 615 nm), enabling multi‐channel and high‐resolution imaging in living cells through the dual‐sensor approach (Figure 4a). The POI in this study included A315E‐Halo, a mutant of the TAR DNA‐binding protein‐43 (TDP‐43) associated with ALS, and H50Q‐Halo, a mutant of the α‐synuclein (SNCA) protein associated with Parkinson disease. In the negative control cells without external stress, the dual sensor exhibited a dark fluorescent background with only blue fluorescence emitted by the cell nuclear dye Hoechst 33342 (Figure 4b, the second panels).

In contrast, in positive control cells treated with 1 μm the proteasome inhibitor MG132 for 24 h, we observed the aggregation of green‐ and red‐emitting punctate fluorescence around the blue‐emitting cell nuclear. After software processing, the overlapping red and green fluorescence formed a yellow signal (Figure 4b, the first panels). These results indicated that under MG132‐induced stress, the sensor‐labeled POI aggregated, creating a sticky microenvironment to activate the sensor fluorescence. The punctate fluorescence corresponded to insoluble protein aggregates, consistent with the results of in vitro fluorescence and turbidity kinetics experiments Furthermore, we performed intracellular experiments to examine POI aggregation under different Zn^2+^ concentrations. High‐resolution imaging of cells treated with 10, 20, and 50 μm Zn^2+^ revealed fluorescence activation consistent with that observed with MG132 treatment (Figure 4b, the last three panels). This demonstrated that Zn^2+^ could promote the formation of insoluble protein aggregates. Additionally, we observed that the protein aggregates enlarged progressively with higher Zn^2+^ concentration, consistent with the in vitro finding that the degree of protein aggregation was positively correlated with the Zn^2+^ concentration.

### 2.4 Protein aggregation causes mitochondrial damage

**FIGURE 5:**
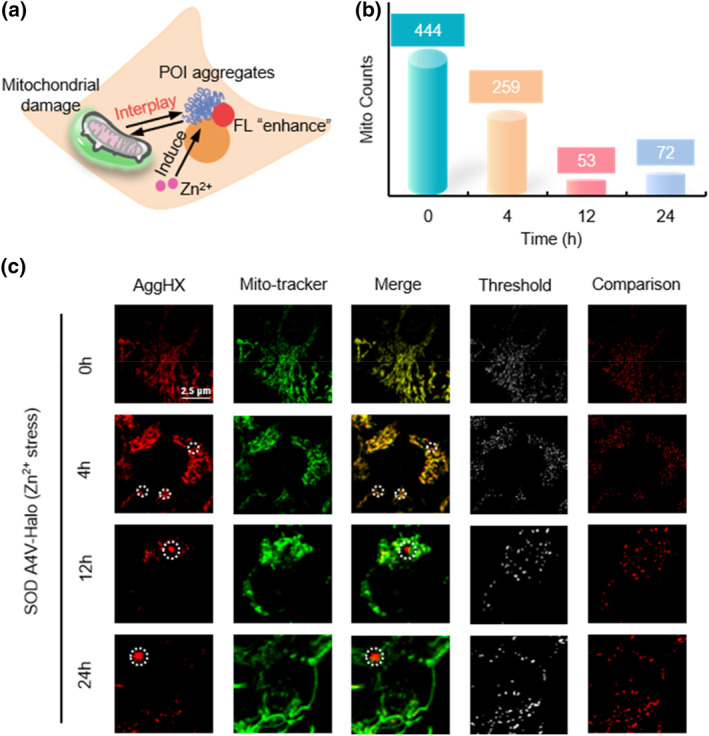
High‐resolution imaging of the interaction between 50 μm Zn^2+^‐induced protein aggregation and mitochondrial homeostatic imbalance. (a) Schematic of AggHX and mitochondrial localization sensors used for imaging protein aggregation and mitochondrial damage. (b) Changes in mitochondrial number in HEK293 cells treated with 50 μm Zn^2+^ for 0, 4, 12, and 24 h. (c) High‐resolution imaging under (b) condition, and mitochondrial count using ImageJ software. Scale bar: 2.5 μm.

Mitochondrial dysfunction is associated with many age‐related diseases, such as cardiovascular‐like diseases and neurodegenerative diseases.[^36^, ^37^, ^38^, ^39^, ^40^] A primary cause of this dysfunction is the misfolding and aggregation of mitochondrial proteins, which become damaged under stress. It has been shown that non‐mitochondrial proteins, constituting pathologic aggregates in neurodegenerative diseases, accumulate around the mitochondria and affect the normal function of mitochondria.[^41^, ^42^, ^43^, ^44^] Mitochondrial dysfunction increases oxidative stress and generates excess reactive oxygen species, further exacerbating protein aggregation and mitochondrial damage, ultimately leading to apoptosis or cell death.[^45^, ^46^, ^47^] Therefore, there is a significant interaction between protein aggregation and mitochondrial damage (Figure 5a).

Cellular experiments demonstrated that 50 μm Zn^2+^ was sufficient to induce protein aggregation, and this concentration did not immediately cause mitochondrial damage (Figure 4b, Supporting Information S1: Figures S6 and S7). Then, 50 μm Zn^2+^ was selected as the inducer for subsequent studies examining the relationship between protein aggregation and mitochondrial dysfunction. HEK293 was co‐stained with AggHX and a green mitochondrial dye (Mito‐tracker), followed by high‐resolution imaging at 0, 4, 12, and 24 h of Zn^2+^ incubation (Figure 5c). Software analysis revealed a gradual decrease in the number of mitochondria with prolonged Zn^2+^ incubation (Figure 5b). High‐resolution imaging at 0 and 4 h showed co‐localization of red and green channels, likely due to the Zn^2+^‐DPA part targeting the anionic phospholipid (phosphatidylserine), which presents in the inner membrane of mitochondria.[^48^, ^49^, ^50^] Only protein aggregates remained in the red channel at 12 and 24 h, facilitating better observation. Along with protein aggregation induced by 50 μm Zn^2+^, mitochondria were gradually damaged, as evidenced by a significant reduction in the number of mitochondria. Protein aggregates gradually enlarged from 4 to 24 h, suggesting that mitochondrial damage further contributed to protein aggregation. In summary, high‐resolution imaging of double‐stained protein aggregates and mitochondria showed a mutually reinforcing relationship between protein aggregation and mitochondrial dysfunction.

### 2.5 FLIM of AggHX in cells

**FIGURE 6:**
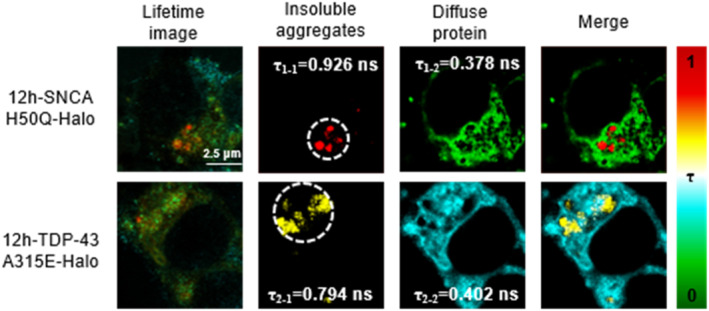
Fluorescence lifetime imaging microscopy imaging of H50Q and A315E protein aggregation induced by 50 μm Zn^2+^. *τ*_1–1_, *τ*_1–2_, *τ*_2–1_, *τ*_2–2_, denoted the average fluorescence lifetime values in the insoluble aggregated state and in the diffuse state of SNCA H50Q‐Halo and TDP‐43 A315E‐Halo, respectively. Scale bar: 2.5 μm.

Eventually, we expressed SNCA H50Q‐Halo and TDP‐43 A315E‐Halo proteins in separate groups of HEK293 cells, both treated with 50 μm Zn^2+^ for 12 h, and performed fluorescence lifetime imaging microscopy (FLIM) on AggHX‐labeled POIs (Figure 6). By analyzing the fluorescence lifetimes of intracellular proteins, we could distinguish between insoluble aggregated particles and diffused proteins in the cytoplasm. The fluorescence lifetime of the AggHX sensor was used to differentiate between these forms: the high fluorescence lifetimes (*τ*_1–1_ and *τ*_2–1_) indicated insoluble protein aggregates, while the low fluorescence lifetimes (*τ*_1–2_ and *τ*_2–2_) corresponded to diffuse proteins. According to in vitro fluorescence lifetime experiment, we roughly analyzed the approximate viscosity value corresponding to the fluorescence lifetime measured in the cells. The viscosity values corresponding to *τ*_1–1_, *τ*_1–2_, *τ*_2–1_, and *τ*_2–2_ are 2455 cP, 26 cP, 813 cP, and 32 cP, which indicate the viscosity variability across different types of POIs treated by Zn^2+^.

## 3 CONCLUSION

In summary, we developed a BODIPY‐based fluorescent sensor (AggHX) with a dual‐response to Zn^2+^ and viscosity. The DPA group in the AggHX selectively complexed Zn^2+^, leading to enhanced red fluorescence. In vitro experiments, we verified that the sensor's fluorescence intensity showed an enhancement trend over a certain Zn^2+^ concentration range and was positively correlated with viscosity. Despite the sensor's fluorescence lifetime being regulated solely by viscosity, it also demonstrated a positive correlation with viscosity. Based on the viscosity response of the AggHX, we monitored the viscosity change of Zn^2+^‐induced neurodegenerative protein aggregation, allowing us to visualize the aggregation process. By combining the AggHX with a mitochondrial dye (green channel), we observed mitochondrial damage resulting from protein aggregation and assessed the impact of this damage on the extent of protein aggregation through high‐resolution imaging. Meanwhile, FLIM of the AggHX sensor in live cells quantified the viscosity values of different POIs after the treatment with the same Zn^2+^ concentration for a fixed duration, revealing varying viscosity levels across different species of protein aggregates. This work provides a reliable tool for the quantitative study of protein aggregation process in cells, especially Zn^2+^‐mediated neurodegenerative proteins, and is significant for the diagnosis and treatment of related diseases.

## CONFLICT OF INTEREST STATEMENT

The authors declare no conflicts of interest.

## ETHICS STATEMENT

No animal or human experiments were involved in this study.

## Supporting information

Supporting Information S1

## Data Availability

The data that support the findings of this study are available from the corresponding author upon reasonable request.
