# Supplementary material for: Exploring the interplay between zinc‐induced protein dyshomeostasis and mitochondrial dysfunction using viscosity‐sensitive sensor
Source: Smart Mol. 2024 Oct 12;2(4):e20240047. doi: 10.1002/smo.20240047 (PMC12118262; doi:10.1002/smo.20240047)
Supplement: Supplementary file 1 — Supporting Information S1 [file SMO2-2-e20240047-s001.docx]

Supporting Information

Exploring the Interplay Between Zinc-induced Protein Dyshomeostasis and Mitochondrial Dysfunction using Viscosity-Sensitive Sensor

*Xuan He^# [a]^, Jiaqi Li^# [a]^, Wenye He^# [c]^, Jia Zhai^[a]^, Yu Wei^[a]^, Xin Zhang^* [b]^, Baoxing Shen**^* [a]^ and He Huang ^[a]^*

^[a]^ X. He, J. Li, J. Zhai, Y. Wei, Prof. B. Shen, Prof. H. Huang

School of Food Science and Pharmaceutical Engineering, Nanjing Normal University, 1 Wenyuan Road, Nanjing 210023, China

E-mail: [shenbx@njnu.edu.cn](mailto:shenbx@njnu.edu.cn)

^[b]^ Prof. X. Zhang

Department of Chemistry, Research Center for Industries of the Future, Westlake University, 600 Dunyu Road, Hangzhou 310030, Zhejiang, China.; Westlake Laboratory of Life Sciences and Biomedicine, 18 Shilongshan Road, Hangzhou 310024, Zhejiang, China

E-mail: [zhangxin@westlake.edu.cn](mailto:zhangxin@westlake.edu.cn)

^[c]^ W. He

Faculty of Science, National University of Singapore, 21 Lower Kent Ridge Road, Singapore 119077, Singapore

^#^ These authors contributed equally.

**Abstract:** Mitochondria are crucial sites for protein quality control within cells. When mitochondrial stress is triggered by protein misfolding, it can accelerate abnormal protein aggregation, potentially inducing various diseases. This study develop a cascade-responsive sensor, named AggHX, to monitor the microenvironment of protein aggregation induced by zinc (II) ions and the accompanying mitochondrial dysfunction. The AggHX consists of two key components: (1) A Zn^2+^ recognition group for trigerring a fluorescent enhance response, and (2) a near-infrared BODIPY scaffold that detects viscosity changes in cell aggregation via HaloTag. This sensor’s mechanism of action is elucidated through photochemical and biochemical characterizations. To further investigate the relationship between protein aggregation and mitochondrial homeostasis, we employ fluorescence lifetime imaging microscopy (FLIM) to assess viscocity changes in protein aggregates under intracellular Zn^2+^ stress. This research provides insights into the dynamic behavior and spatial distribution of protein aggregates and mitochondria, contributing to a deeper understanding of their physiological roles in cellular processes and potential implications in disease pathology.

Table of Contents

1. **Experimental methods**

1.1 Plasmids

1.2 Protein expression and purification

1.3 Absorbance and fluorescence spectra measurement

1.4 Metal ion experiments

1.5 Viscosity sensitivity measurement and calculation

1.5 Interference experiments

1.7 *In vitro* protein aggregation experiments

1.8 High-resolution imaging

1.9 FLIM

1. **Supplementary Figures**

2.1 Spectrogram

2.2 Limit of Detection and Zn^2+^ Titration Plateau

2.3 Anion independence experiments

2.4 AggHX fluorescence lifetime response to viscosity

2.5 Temperature test

2.6 Imaging of mitochondrial damage

2.7 Stacked Histogram of mitochondrial damage

1. **Synthetic Methods**

3.1 Synthesis of BODIPY fluorescent nucleus

3.2 Synthesis of metal identify groups

3.2 Synthesis of Halo-linker

3.3 Synthesis of AggHX

1. **H/C NMR and HRMS spectras**
2. **NMR and HRMS Characterizations**
3. **Experimental Methods**

For the following *in vitro* tests, the cuvette mode of the HITACHI F-7100 fluorescence spectrophotometer was used to detect fluorescence intensity, and the LS-50B molecular fluorescence spectrometer was used to detect fluorescence lifetime, unless otherwise specified.

In cellular experiments, high-resolution imaging was conducted with the Airyscan mode of the ZEISS LSM 980, and FLIM imaging was carried out using the LEICA STELLARIS 8 FALCON.

**1.1 Plasmids**

Mammalian expression: pHTN vector (Promega, Inc) with a stop codon added to the C-terminal of Halo-Tag. The mutations were created by QuickChange PCR.

Protein expression: pET29b vectors were constructed to encode Halo-Tag.

**1.2 Protein Expression and Purification**

Protein expression: Halo Tag, SOD1 A4V-Halo: Plasmids were transformed into E. coli BL21DE3* competent cells harboring a pBAD vector encoding σ32-I54N. Successfully transformed E. coli was inoculated into liquid medium containing Kanamycin (Kan), and the culture was expanded to OD_600_=0.6~0.8. Subsequently, Isopropyl-d-1-thiogalactopyranoside (IPTG) was added to induce protein expression overnight at 18°C. In brief, cells expressing recombinant proteins were thawed and lysed by sonication at 4ºC in buffer A (50 m**m** Tris-HCl, pH 7.5, 100 m**m** NaCl) with addition of a protease inhibitor (1 m**m** PMSF). Lysed cells were centrifuged at 8000 rpm for 60min at 4ºC. The supernatant was collected through a 0.22 μm filter membrane and loaded onto a BioRad Nuvia Ni-IMAC column (6 mL) and washed with buffer A. The protein was then eluted by gradient addition of buffer B containing Tris • HCl (50 m**m,** pH 7.5), NaCl (100 m**m**), and imidazole (500 m**m)**. The protein fractions were identified by SDS-PAGE analysis, pooled, and concentrated. After that, the gel filtration column (120 mL HiPrepTM 16/60 SephacrylTM S200 HR) was used to allow further protein purification and buffer exchange (50 mM Tris-HCl, pH 7.5, 100 mM NaCl). No significant impurities were identified and purity was estimated to be > 98% based on SDS-PAGE.

**1.3 Absorbance and Fluorescence Spectra Measurement**

Spectroscopic measurements were obtained from a sensor (20 mm) and Zn^2+^ solution (200 μm), prepared in a 50:50 mixture of ethanol (EtOH) and ultrapure water (ddH_2_O). The sensor was thoroughly mixed, and the sample (200 μL) was transferred to a quartz spectrophotometer cell for absorption spectrum analysis, which was recorded using an ultraviolet spectrophotometer (Cary 5000). Another aliquot of the sample (200 μL) was placed in a quartz fluorescent spectrophotometer cell, and the excitation and emission spectra were recorded using a HITACHI F-7100 fluorescence spectrophotometer. Unless otherwise specified, all subsequent fluorescence intensity measurements were performed using the cuvette mode of the HITACHI F-7100 fluorescence spectrophotometer.

**1.4 Metal Ion Experiments**

Ion selective test: A sensor (20 μm) and metal ion solution (200 μm) in the mixture of 50% EtOH and 50% ddH_2_O. After thorough mixing, the fluorescence intensity was measured in the HITACHI F-7100 fluorescence spectrophotometer. The fluorescence intensity of solution with Zn^2+^ was normalizated to 1.

Ion titration experiment: The fluorescence intensity of AggHX (20 μm) in the Zn^2+^ range of 1-50 equivalent (eq) was measured using a HITACHI F-7100 fluorescence spectrophotometer. The fluorescence lifetime of the sensor (5 μm) was measured at Zn^2+^ concentrations of 10, 30, 40, 50, 60, and 70 eq by the LS-50B molecular fluorescence spectrometer. The fluorescence intensity and fluorescence lifetime at 50 eq Zn^2+^ were normalized to 1, respectively. The standard deviation (SD) of the fluorescence intensity of sensor without Zn^2+^ was calculated for ten times. The linear fitting of the fluorescence intensity to Zn^2+^ concentration was performed in the titration experiment, and the slope of the trend line was k. Limit of detection (LOD) of AggHX for Zn^2+^ was calculated by following formula:

LOD = 3S/k

Where, S represents the standard deviation and k represents slope of the titration fitting line.

Job plot: Solutions with varying concentrations of Zn²⁺ and AggHX were prepared, maintaining a total concentration of 100 μm. The fluorescence response of the sensor was measured across a Zn^2+^ concentration gradient ranging from 10% to 90%. The fluorescence intensity of the mixture containing Zn^2+^ (70 μm) and sensor (30 μm) was normalized to 1. Error bar was calculated from three-time independent experiments.

Fluorescence kinetics of Zn^2+^-induced of AggHX: AggHX (20 μm) was dissolved in a mixture (200 μL) of EtOH/ddH_2_O=1:1, followed by the drop addition of Zn^2+^ (80 μm), and the fluorescence kinetics was rapidly measured using the Bio Tek Synergy H1 microplate reader. Error bar was calculated from three-time independent experiments.

**1.5 Viscosity Sensitivity Measurement and Calculation**

Fluorescence intensity: All sensors (20 μm) were prepared in a series of ethylene glycol/glycerol (EG/G) solution in the following mixing ratios: EG/G = 70/30 (81 cP), 50/50 (183 cP), 40/60 (283 cP), 30/70 (426 cP), 20/80 (621 cP) and 100% G (1069 cP). Then, the fluorescence was recorded on Tecan infinite M1000Pro fluorescence microplate reader. The fluorescence intensity in 80% glycerol was normalized to 1. All the fluorescence intensity was divided by the intensity in 80% glycerol. Error bar was calculated from three-time independent experiments. A logarithm plot of emission intensity as a function of viscosity was used to determine the value of viscosity sensitivity ($x$) based on the Förster-Hoffmann equation $logI=xlog\eta+C$, wherein $\eta$ is viscosity (cP), $I$ is the fluorescence intensity, $x$ is the viscosity sensitivity. The viscosity test for AggHX + Zn^2+^ was conducted under the same conditions as above, except that Zn^2+^ (120 eq) was added.

Fluorescence lifetime: The fluorescence lifetime measurements were conducted similarly to the viscosity response experiment for fluorescence intensity, with the only difference being that a sensor (10 μm) solution was used.

**1.6** **Interference Experiments**

pH: The fluorescence of sensor (20 μm), both alone and in combination with Zn²⁺ (400 μm), was measured across a pH range from 5 to10. The fluorescence intensity of AggHX + Zn^2+^ at the pH=10 system was used to normalized. Error bar was calculated from three-time independent experiments.

Polarity: The uncomplexed sensor (20 μm) was mixed in a series of methanol (MeOH)/dioxane mixtures with MeOH ratios of 20%, 30%, 40%, 50%, and 60%. The fluorescence intensity in the 20% MeOH system was normalized to 1, all other fluorescence intensities were divided by the intensity in the 20% MeOH system. Error bar was calculated from three-time independent experiments.

Anion: The solution of anion (10 mm) including SO_4_^2-^, OH^-^, HCO_3_^-^, CO_3_^2-^, H_2_PO_3_^-^, Cl^-^ was prepared using ddH_2_O. The stock solution of AggHX and Zn²⁺ (1:20 eq) was prepared at a concentration of AggHX (10 μm) and Zn²⁺ (200 μm) in a 3:20 mixture of EtOH and ddH₂O, and this mixture (200 μL) was used. Subsequently, the anion (100 μm) was added and the fluorescence intensity was measured. The maximum fluorescence intensity was used for normalization. The anion experiment for uncombined sensor was conducted as above, by replacing the stock solution of AggHX + Zn^2+^ (1:20 eq) with AggHX (10 μm). Error bar was calculated from three-time independent experiments.

**1.7 *In vitro* Protein Aggregation Experiments**

Buffer condition: All Protein experiments were tested in a buffer including tris-HCl (50 mm), NaCl (100 mm), and EDTA (80 mm) at pH 7.5.

Temperature test: In the presence or absence of Zn^2+^ (20 mm), fluorescence intensity was measured immediately after incubating the mixture of sensor (10 μm) and SOD A4V-Halo (40 μm) for 6min at 25°C and 59°C, respectively. The fluorescence intensity of AggHX + Zn^2+^ at 59°C was normalized to 1, and all other fluorescence intensities were divided by the fluorescence intensity. Subsequently, a temperature gradient ranging from 25°C to 59°C was applied to incubate the sensor and protein mixture for 6min, and the fluorescence intensity was measured to plotted a histogram. Error bar was calculated from three-time independent experiments.

Zn^2+^ induce protein aggregation: A series of Zn^2+^ concentrations (3, 4, 5, 6, 7, 8, 9 mm) were added to a mixture of AggHX (20 μm) and A4V mutant (60 μm), and the change in fluorescence intensity was recorded. The fluorescence intensity under the Zn^2+^ (9 mm) condition was used for normalization.

Fluorescence intensity and turbidity kinetics: Zn^2+^ (800 μm) was added to a mixture of AggHX (10 μm) and A4V mutant (40 μm), and the kinetics of fluorescence intensities were rapidly measured using the Bio Tek Synergy H1 microplate reader. The absorption at 330 nm was measured under the same conditions in the parallel group to describe the turbidity kinetics. Error bar was calculated from three-time independent experiments.

**1.8** **High-Resolution Imaging**

The HEK293 cells were seeded at 25% confluency 24h prior to transfection in poly-D-lysine coated 20 mm glass bottom dishes. Cells were grown in DMEM media supplemented with 10% FBS and Penicillin-Streptomycin antibiotics until they reached 50-60% confluency. Transfection was carried out using X-tremeGene™ 9 DNA transfection reagent (Roche) according to the manufacturer’s instructions. Proteins were expressed for 24h prior to analyses. To label proteins with Halo-Tag fusion, protein expression was carried out in the presence of AggHX (1 μm) and GFP-Halo (2 μm) to form covalent conjugate with the Halo-Tag or domain after treatment of cells with different Zn^2+^ concentrations for 2h. The positive control group was treated with MG132 (2 μm) for 24h. Cells were stained with Hoechst 33342 (5 μm) and incubated at 37°C for 20min. After incubation, excess probes and unbound Halo ligands were washed off with PBS and DMEM. Finally, added 2 mL DMEM and stabilize in a 37°C incubator for 30min before imaging. Hight-resolution images were obtained using ZEISS LSM 980 microscope. AggHX was visualized using the red laser (561 nm, GFP-Halo was visualized using the green laser (488 nm), and Hoechst 33342 was visualized using the blue laser (405 nm). Moreover, mitochondrial staining used commercial Mito-tracker green (Thermo Fisher) (1 μm) to incubate 30min and was visualized using the green laser (488 nm).

To determined the concentration of Zn^2+^ damage mitochondria, cells were treated with a series of Zn^2+^ Concentrations (0, 20, 50, 100 and 200 μm) for 1h and then stained with JC-1 (5 μm) for 30min. High-resolution imaging was performed using the LEICA STELLARIS 8 FALCON. The maximum excitation wavelength of damaged mitochondria for imaging was 514 nm, while that of normal mitochondria for imaging was 585 nm.

For all imaging experiments, laser intensities, gain and other settings were kept identical to exclude artifacts and ensure robustness of data.

**1.9 FLIM**

The cell culture and staining processes were consistent with the part of high-resolution imaging (part of 1.8). Fluorescent lifetime signals were recorded by LEICA STELLARIS 8 FALCON.

1. **Supplementary Figures**

**2.1 Spectrogram**


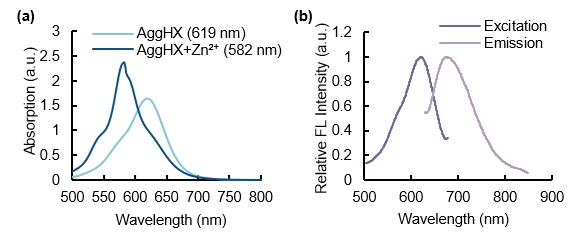


Figure S1. (a) Absorption spectrum of AggHX and AggHX/Zn^2+^. (b) Excitation and emission spectra of AggHX. Λ_ex_ = 621 nm; λ_em_ = 674 nm.

**2.2 Limit of Detection and Zn^2+^ Titration Plateau**

**
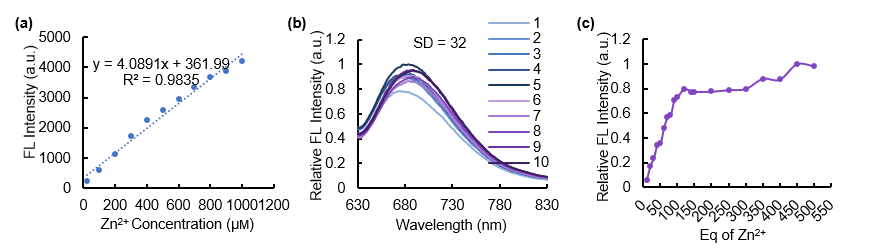
**

**Figure S2.** (a) The linear fitting of the fluorescence intensity to Zn^2+^ concentration. (b) Ten replicate measurements of the fluorescence intensity of AggHX without complexed Zn^2+^. (c) Fluorescence intensity of AggHX (20 μm) sensor at different concentrations of Zn^2+^. At 120 eq Zn^2+^ (2.4 mm), sensor’s fluorescence intensity entered the plateau phase, and did not change with Zn^2+^ concentration.

**2.3 Anion Independence Experiments**

**
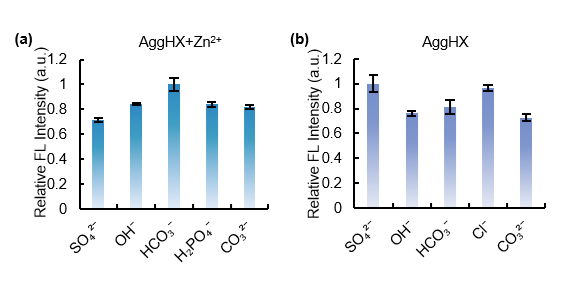
**

Figure S3. Stability of the AggHX sensor in the presence or absence of Zn^2+^ for the anion. Error bars: standard error (n = 3).

**2.4** **AggHX Fluorescence Lifetime Response to Viscosity**


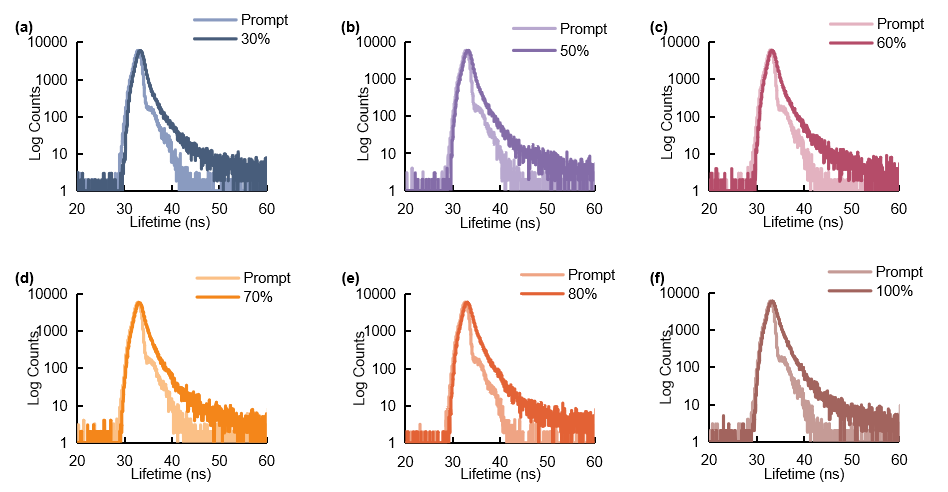


Figure S4. Fluorescence lifetimes of AggHX (10 μm) and Zn^2+^ (200 μm) in mixed EG/G solutions at different ratios. The ratio of G ranges from 30% to 100%, and the corresponding viscosity of the system is 81 cP, 183 cP, 283 cP, 426 cP, 621 cP and 1069 cP, respectively.

**2.5 Temperature Test**


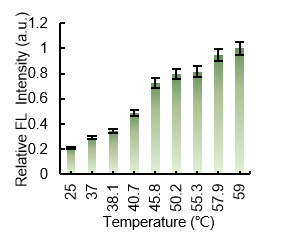


Figure S5. Temperature gradients induced SOD A4V-Halo aggregation. the protein began to aggregate at 45.8℃ and the protein completely formed insoluble aggregates at 57.8-59℃. Error bars: standard error (n = 3).

**2.6 Imaging of Mitochondrial Damage**

**
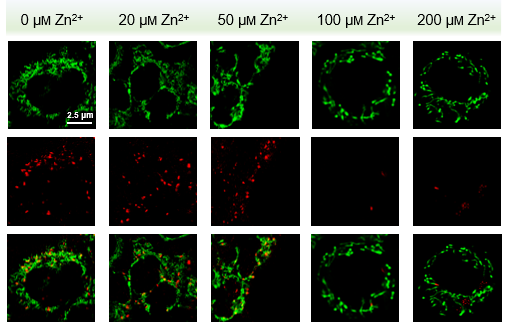
**

Figure S6. JC-1 (1 μm) stained Zn^2+^ concentration gradient-treated HEK293 cells. Green: damaged mitochondria (λ_ex_ = 514 nm, λ_em_ = 529 nm), red: normal mitochondria (λ_ex_ = 585 nm, λ_em_ = 590 nm). Scale bar: 2.5 μm.

**2.7 Stacked Histogram of Mitochondrial Damage**

**
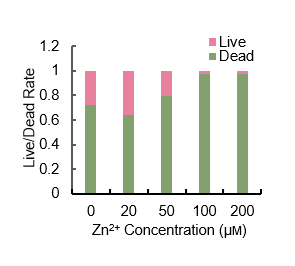
**

Figure S7. Stacked Histogram of normal and dead mitochondria at different Zn^2+^ concentrations in Figure S5.

**3.** **Synthetic Methods**

**3.1 Synthesis of BODIPY Fluorescent Nucleus**


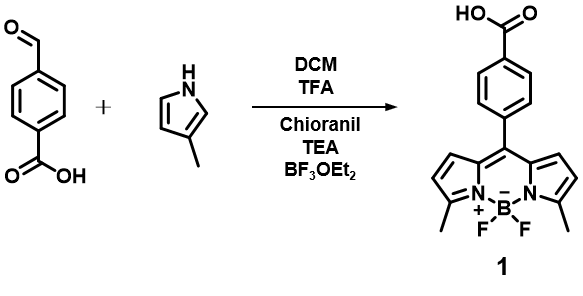


Scheme S1. Synthetic of compound 1

Compound 1: 4-Formylbenzoic acid (1 eq) and 2-methyl-1H-pyrrole (2 eq) were dissolved in dichloromethane (DCM) (40 mL), 1-2 drops of trifluoroacetic acid (TFA) was added as a catalyst and the reaction mixture was stirred at room temperature overnight. The next day, tetrachlorobenzoquinone (1 eq) was added in batches. The triethylamine (TEA) (10 mL) was added to the reaction system after 6h, and boron trifluoride ethyl ether (BF_3_OEt_2_) (1 eq) was added after sufficient reaction and stirred overnight. At the end of the reaction, the reaction was quenched with water and the product was extracted using DCM. The organic phase was removed under reduced pressure and separated and purified using column chromatography to give a red solid powder, and the eluent ratio was petroleum ether (PE): DCM: EtOH = 5:5:1).

**3.2 Synthesis of Metal Identify Groups**


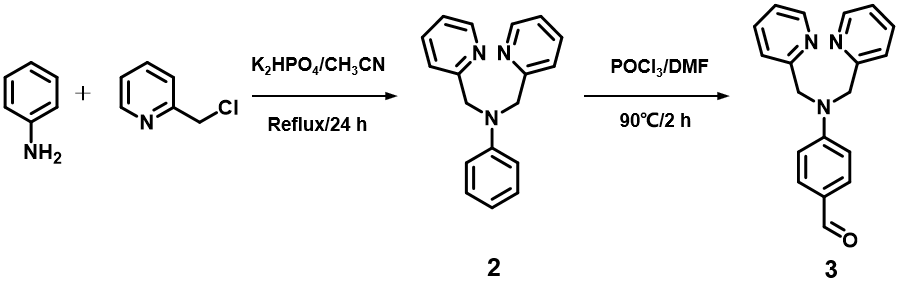


Scheme S2. Synthetic of compound 2 and compound 3

Compound 2: aniline (0.931 g, 10.0 mm) and K_2_HPO_4_ (5.22 g, 30.0 mm) were dissolved in acetonitrile (CH_3_CN) (30 mL) and chloromethyl pyridine hydrochloride (4.59 g, 28.0 mm) was added drop by drop at 0°C (dissolved in 1 mL of water ahead of time). The mixture was stirred for 30min at room temperature. The reaction mixture was refluxed at 90°C for 24h and dot-plate monitoring was performed. At the end of the reaction, the reaction was quenched by adding a few milliliters of water to the reaction mixture and extracted with ethyl acetate (EA). This intermediate was purified by silica gel column chromatography (DCM: EA = 3:1) to give a yellowish brown crystalline solid.

Compound 3: phosphorus oxychloride (POCl_3_) (2 mL) was added dropwise to N, N-Dimethylformamide (DMF) (4 mL) and stirred in an ice-water bath for 30min. Compound 2 (1 g) was dissolved in DMF (1.5 mL), added dropwise to the reaction system, and heated at 90°C for 2h. Upon completion of the reaction, which resulted in an oily-like product, the reaction mixture was cooled to room temperature, followed by the addition of ice-water and solid sodium acetate. The reaction mixture was stirred and the pH was monitored using pH paper until it was neutralized to pH 7. The mixture was then extracted with DCM and dried with anhydrous sodium sulfate. Finally, a yellow oily product was obtained by silica gel column chromatography (DCM: EA = 1:1, 2,4-dinitrophenylhydrazine color developed)

**3.3 Synthesis of Halo-Linker**


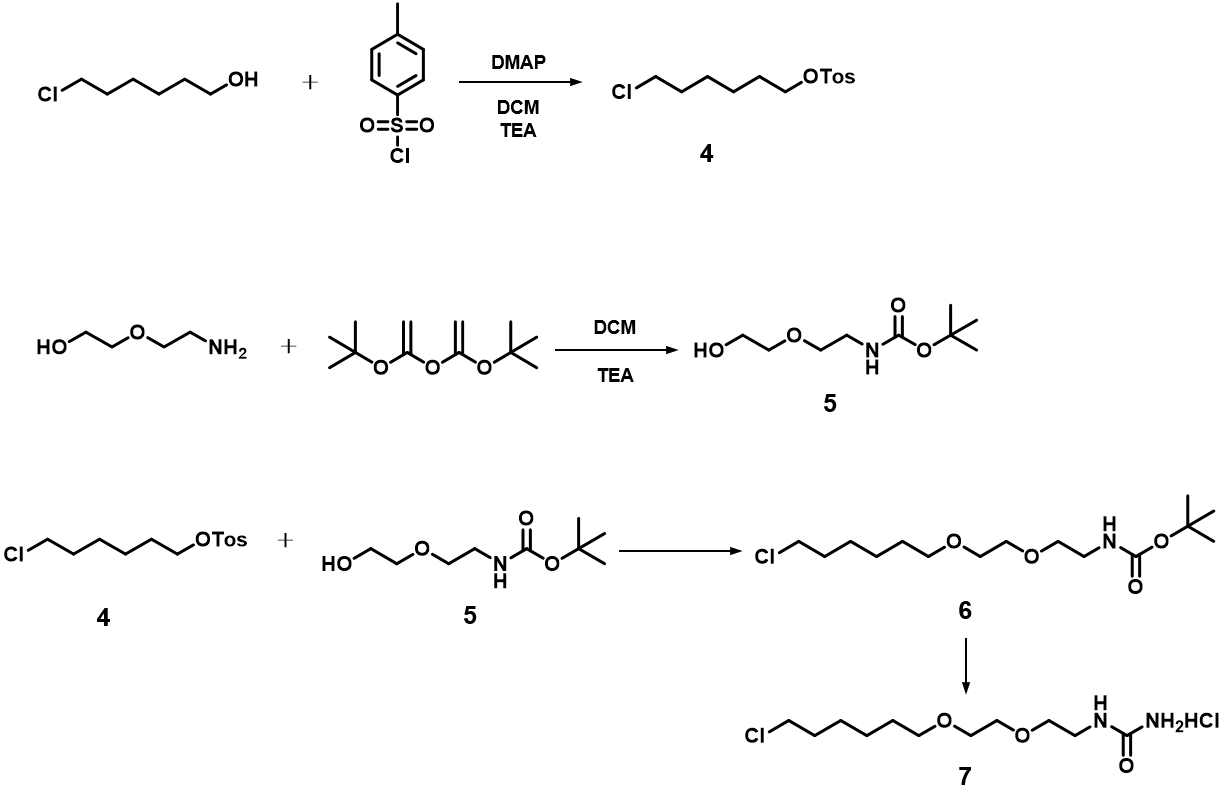


Scheme S3. Synthetic of compound 7

Compound 4: p-toluenesulfonyl chloride (7.5 g) and 6-chloro-1-hexanol were dissolved (2.4 mL) in DCM (20 mL) and stirred for 20min. 4-dimethylaminopyridine (DMAP) (2.235 g) was added and TEA (8-10 mL) was added dropwise, which appeared as a solidified gel. The reaction was monitored by thin layer chromatography (TLC) after 2h. The organic solvent was removed by rotary evaporation and purified by column chromatography (PE: EA= 8:1) to obtain a white oily substance.

Compound 5: diethylene glycol amine (1.505 mL) was dissolved in DCM, and di-tert-butyl dicarbonate (3.726 g) was dissolved into DCM (9 mL). This solution was was added to the reaction system drop by drop within 25min. The reaction was completed for 6h, and the plate was stained with ninhydrin (PE: EA = 2:1), get the product for oily substances.

Compound 6: Pre-prepared potassium tert-butanol (1m) formulated with tetrahydrofuran was pre-cooled to 4°C. Compound 5 (0.7 g) was dissolved in N,N-dimethylformamide (6 mL) and the prepared potassium tert-butanolate was added. After half an hour of reaction, compound 4 (1 g) was added drop wise. The reaction was carried out overnight at room temperature, the product was color developed with ninhydrin and purified by column chromatography (PE: EA = 3:1), yielding a clear oily liquid.

Compound 7: Compound 6 (0.24 g) was dissolved in Dioxane-HCl (2 mL) and stirred at room temperature for 1h for deprotection, with progress monitored using TLC (PE: EA = 1:1). At the end of the reaction, the reaction was quenched with DCM (20 mL) and evaporated under reduced pressure to remove the organic solvent, yielding compound 7.

**3.4 Synthesis of AggHX**

Compound 8: A 50 mL bottle was selected to dissolve compound 1 (1 eq) and compound 7 (1.2 eq) in DMF (4 mL), followed by ethyl [3-(dimethylamino) propyl] carbodiimide hydrochloride (EDC·HCl) and 1-hydroxybenzotriazole monohydrate (HOBt·H_2_O). Subsequently, TEA was added. The reaction mixture was carried out under overnight conditions. After the reaction, the mixture was extracted with DCM and water. The organic phase was then dried over anhydrous sodium sulfate and purified by column chromatography (PE: DCM: EtOH = 16:16:1), yielding a red powder solid.

AggHX: Compound 8 (1 eq) is dissolved in toluene (10 mL) under a nitrogen protection. Compound 3 (2 eq) was added, and the mixture was heated with stirring. Once the temperature reached 50°C, p-toluenesulfonic acid (TsOH) (1.5 eq) was added, followed by the dropwise addition of piperidine. The temperature was raised to 110°C, and a condenser tube and oil/water separator were installed to prevent solvent evaporation and by-products formation. The reaction was completed by the appearance of a red fluorescent substance, on TLC. The toluene solution was removed using a rotary evaporator. The organic phase was extracted with DCM and water, then dried with anhydrous sodium sulfate. Final purification by column chromatography yielded a dark green solid powder.


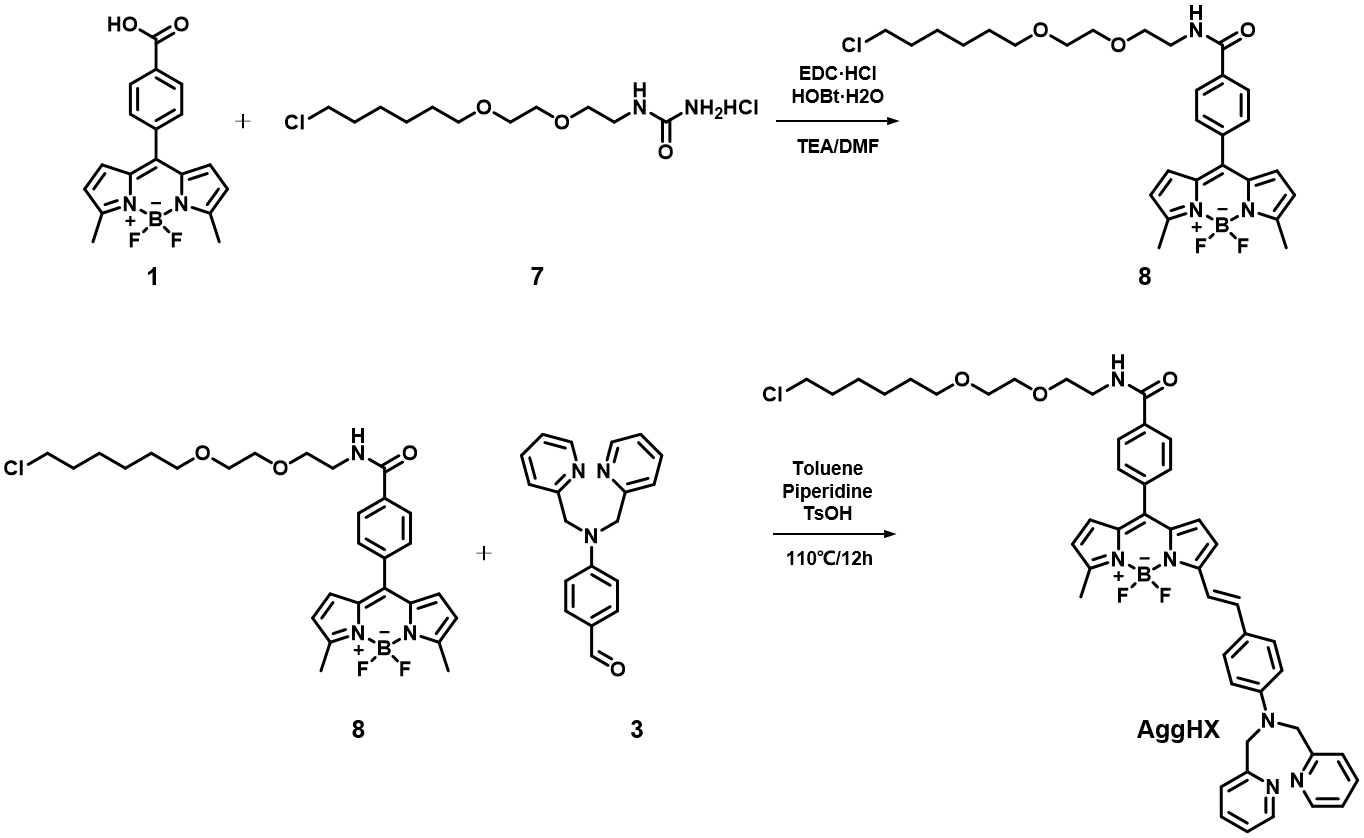


Scheme S4. Synthetic of AggHX

**4. H/C NMR and HRMS Spectras**


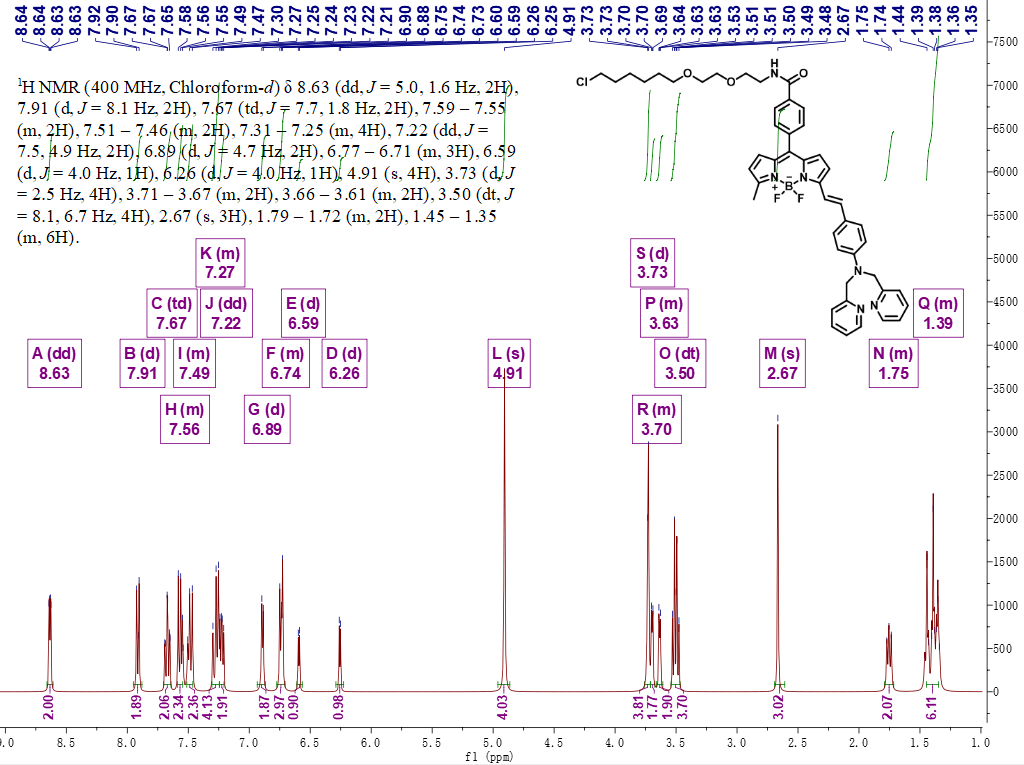


Figure S8. ^1^H-NMR of AggHX.


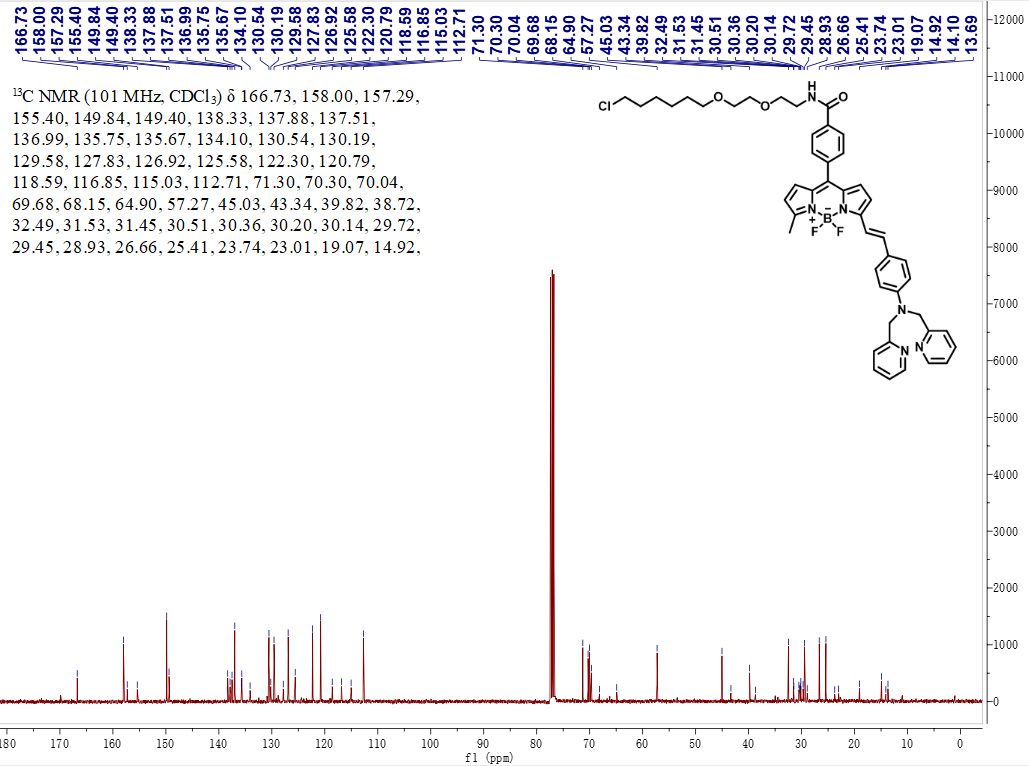


Figure S9. ^13^C-NMR of AggHX.


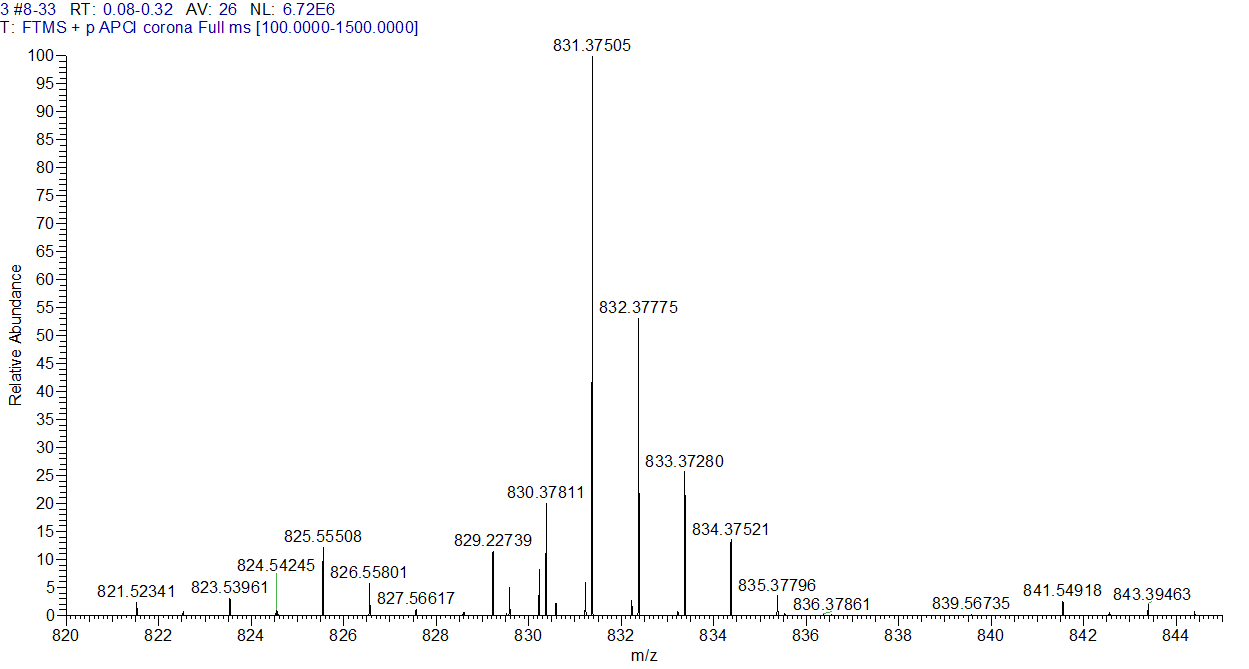


Figure S10. HRMS of AggHX in MeOH.

1. **NMR and HRMS Characterizations**

AggHX: (E)-4-(3-(4-(bis(pyridin-2-ylmethyl)amino)styryl)-5,5-difluoro-7-methyl-5H-5l4,6l4-dipyrrolo[1,2-c:2',1'-f][1,3,2]diazaborinin-10-yl)-N-(2-(2-((6-chlorohexyl)oxy)ethoxy)ethyl)benzamide

The ^1^H-NMR Characterization of AggHX: ^1^H NMR (400 MHz, Chloroform-d) δ 8.63 (dd, J = 5.0, 1.6 Hz, 2H), 7.91 (d, J = 8.1 Hz, 2H), 7.67 (td, J = 7.7, 1.8 Hz, 2H), 7.59 – 7.55 (m, 2H), 7.51 – 7.46 (m, 2H), 7.31 – 7.25 (m, 4H), 7.22 (dd, J = 7.5, 4.9 Hz, 2H), 6.89 (d, J = 4.7 Hz, 2H), 6.77 – 6.71 (m, 3H), 6.59 (d, J = 4.0 Hz, 1H), 6.26 (d, J = 4.0 Hz, 1H), 4.91 (s, 4H), 3.73 (d, J = 2.5 Hz, 4H), 3.71 – 3.67 (m, 2H), 3.66 – 3.61 (m, 2H), 3.50 (dt, J = 8.1, 6.7 Hz, 4H), 2.67 (s, 3H), 1.79 – 1.72 (m, 2H), 1.45 – 1.35 (m, 6H).)

The ^13^C-NMR Characterization of AggHX: ^13^C NMR (101 MHz, Chloroform-d) δ 166.73, 158.00, 157.29, 155.40, 149.84, 149.40, 138.33, 137.88, 137.51, 136.99, 135.75, 135.67, 134.10, 130.54, 130.19, 129.58, 127.83, 126.92, 125.58, 122.30, 120.79, 118.59, 116.85, 115.03, 112.71, 71.30, 70.30, 70.04, 69.68, 68.15, 64.90, 57.27, 45.03, 43.34, 39.82, 38.72, 32.49, 31.53, 31.45, 30.51, 30.36, 30.20, 30.14, 29.72, 29.45, 28.93, 26.66, 25.41, 23.74, 23.01, 19.07, 14.92, 14.10, 13.69.)

The HRMS Characterization of AggHX: [M+H]^+^ Calcd, 831.3694, Obsd, 831.3751.

# Author Contributions

B.S. thanks support from the Qing Lan Project of Jiangsu Province, National Natural Science Foundation of China (Grant No. 22007048), Natural Science Foundation of Jiangsu Basic Research Program (BK20221324). In addition, Xuan He, Jiaqi Li and Wenye He contributed equally to this work.
